# Rötheln or German Measles

**Published:** 1911-05-13

**Authors:** 


### Diseases of Children.

### RÖTHELN OR GERMAN MEASLES.

Although the affection known under the names of German measles, rötheln, or rubella, is one which usually runs a mild and harmless course, its diagnosis is a matter worth most careful consideration. The importance of excluding a mild attack of scarlet fever cannot be too greatly insisted upon, not only in view of the necessity for more prolonged treatment, but also on account of the need for the isolation of the patient. It is unnecessary to dwell on the liability to grave complications associated with even the mildest forms of scarlet fever, or on the frequently observed fact that severe attacks of that disease may be propagated from quite slight cases. Similar remarks may be applied to the differentiation of rötheln from measles.

The clinical features of German measles are almost always of a slight character, and were it not for the rash it is probable that many patients would not come under the observation of a medical man at all. This is, indeed, one of the most valuable points in the diagnosis, seeing that in both measles and scarlet fever important and definite symptoms are much more commonly to be met with. Attention, then, being first directed to the patient on account of the rash, it is well here to consider the features of the eruption of German measles

### The Rash.

The typical eruption is distributed from the head and face downwards, and consists of very slightly elevated spots, more macular than papular, generally smaller than those of measles and of a redder colour, without the bluish tinge commonly present in the ordinary measly rash. In rötheln the fine punctate rash as seen in a typical case of scarlet is never to be observed; nor does the region about the mouth escape invasion by the eruption. This latter point is of great importance in the diagnosis, for it may be said that rötheln is more likely to be confused with mild scarlet than with measles, because in the former the rash, appearing as it does about the second day, may not be preceded by any very marked symptoms in slight cases, while in the case of measles the period preceding the rash is more often accompanied by some of the prodroma, such as sneezing, lachrymation, or cough, not to mention the assistance afforded by the presence of Koplik's spots in coming to a conclusion.

The rash is essentially a variable one, now resembling scarlet-fever types rather than measles, now looking more like a fading morbilliform rash. It spreads irregularly, though it usually becomes first visible on the face and neck and may be more marked on areas of the skin which have been subjected to pressure. The individual macules of rötheln are not so coarse as those which occur in measles, and in some parts of the body are so far apart sometimes that the normal character of the intervening skin can be definitely determined. The rash is normally of a pale type, never brilliant and vivid like many cases of scarlet; it may also appear and disappear irregularly over the surfaces, so that a fresh patch may make its appearance just as a patch in some other region is fading.

As regards the fauces and tongue, there is a notable absence of that intense injection and swelling of the tonsils and soft palate so common in scarlet fever, while the tongue in German measles is usually only lightly charged and the characteristic prominent papillæ are absent. There is generally some reddening of the faucial structures, but practically no swelling. The conjunctivæ may be moderately injected, but this is never so marked as in the " bleary-eyed " suffusion of typical measles, nor is there present that photophobia so frequent in the latter disease.

### Some Diagnostic Signs.

Considerable stress is usually laid on the presence at a very early stage of definite enlargement of the superficial cervical glands, particularly of those in posterior triangles of the neck and of the suboccipital glands. This is an occurrence frequently associated with German measles and has been sometimes observed before the appearance of the rash itself. Care must be taken to exclude glandular enlargement due to verminous heads, sores on the scalp, or a specific adenitis. If carefully looked for, enlargement of the glands in the axillary and inguinal regions also may be found occasionally in this disease. The swelling soon subsides and the glands do not suppurate.

The temperature in rötheln is not often raised above 100° or 101° and it comes down in two or three days. The patient as a rule has little or no discomfort or malaise and convalescence is rapid. Complications are exceedingly rare, and from a practical point of view they may be disregarded or looked upon as coincidences. The occurrence of albuminuria should arouse suspicion of the correctness of the diagnosis.

As in other eruptive fevers, there is a stage of desquamation; in German measles this is often overlooked, as the flakes thrown off are very fine, branny particles. It may be here mentioned that the duration of the rash of rötheln is very variable, from a few hours to ten or fifteen days. This may be of some assistance in diagnosis, for the rash of scarlet fever lasts rarely for more than four or five days, or that of measles for more than a week.

It will be seen that in arriving at a diagnosis one has to a certain extent to depend upon negative observations, such as the absence of catarrhal signs in the nose and lungs, the slightness and short duration of the pyrexia and the absence of sore throat. Attention has been called to an eruption on the soft palate and uvula as an aid in the diagnosis, but the characteristics of the spots described are hardly sufficiently distinctive to warrant the placing of much reliance on this feature, unless exceptional opportunities are available, as in epidemics.

The length of the incubation period—namely, from about twelve to twenty-one days, will often be of assistance in coming to a conclusion.

